# Gsw-fi: a GLM model incorporating shrinkage and double-weighted strategies for identifying cancer driver genes with functional impact

**DOI:** 10.1186/s12859-024-05707-8

**Published:** 2024-03-06

**Authors:** Xiaolu Xu, Zitong Qi, Lei Wang, Meiwei Zhang, Zhaohong Geng, Xiumei Han

**Affiliations:** 1 School of Computer and Artificial Intelligence, Liaoning Normal University, Dalian, China; 2 Department of Statistics, University of Washington, Seattle, USA; 3 Center for Reproductive and Genetic Medicine, Dalian Women and Children’s Medical Group, Dalian, China; 4 Department of Cardiology, Second Affiliated Hospital of Dalian Medical University, Dalian, China; 5 College of Artificial Intelligence, Dalian Maritime University, Dalian, China

**Keywords:** Cancer research, Driver gene, Mutation functional impact, Generalized linear regression model, Statistical method

## Abstract

**Background:**

Cancer, a disease with high morbidity and mortality rates, poses a significant threat to human health. Driver genes, which harbor mutations accountable for the initiation and progression of tumors, play a crucial role in cancer development. Identifying driver genes stands as a paramount objective in cancer research and precision medicine.

**Results:**

In the present work, we propose a method for identifying driver genes using a Generalized Linear Regression Model (GLM) with Shrinkage and double-Weighted strategies based on Functional Impact, which is named GSW-FI. Firstly, an estimating model is proposed for assessing the background functional impacts of genes based on GLM, utilizing gene features as predictors. Secondly, the shrinkage and double-weighted strategies as two revising approaches are integrated to ensure the rationality of the identified driver genes. Lastly, a statistical method of hypothesis testing is designed to identify driver genes by leveraging the estimated background function impacts. Experimental results conducted on 31 The Cancer Genome Altas datasets demonstrate that GSW-FI outperforms ten other prediction methods in terms of the overlap fraction with well-known databases and consensus predictions among different methods.

**Conclusions:**

GSW-FI presents a novel approach that efficiently identifies driver genes with functional impact mutations using computational methods, thereby advancing the development of precision medicine for cancer.

## Background

Cancer is a fatal disease caused by the accumulation of mutations throughout an individual’s life [1]. Driver mutations are essential for the manifestation of cancer characteristics, whereas passenger mutations, which are random mutations occurring in the background, do not contribute to tumor development and arise during DNA replication [2]. Next-generation sequencing (NGS) technology has revolutionized cancer research by providing a new perspective. Genomic sequencing data encompassing major cancer types are readily accessible through various cancer sequencing projects, including The Cancer Genome Atlas (TCGA) [3] and the International Cancer Genome Consortium (ICGC) [4]. Distinguishing cancer-associated genes with driver mutations, which confer a selective advantage during tumor development, remains an immense challenge despite the availability of reliable and valuable sequencing data. The unequivocal identification of driver genes not only enhances the understanding of tumor progression and also guarantees the effectiveness of gene-targeted cancer therapy [5].

Numerous methods have been developed to identify cancer driver genes by leveraging multi-omics data. In general, these methods can be categorized into three groups. The first category consists of traditional frequency-based methods, which identify genes exhibiting a significantly higher mutation frequency than expected across multiple tumor samples [6–8]. MuSiC [6] and MutSigCV [7] are two frequency-based methods that have been used widely [9–11].MuSiC utilizes various statistical methods to distinguish significant events from passenger mutations, offering a comprehensive, data-driven statistical analysis of NGS datasets. MutSigCV constructs a mathematical model to calculate the gene-specific background mutation rate based on mutational heterogeneity, effectively reducing the inclusion of implausible genes. However, frequency-based methods are limited by their inability to identify driver genes with low population mutation frequencies [12–15].

The second category is comprised of function-based methods that assess the functional impact of mutations by leveraging evolutionary information [16–20]. These methods identify genes that exhibit a significant bias towards accumulating high-impact mutations. For instance, e-Driver identifies potential cancer driver genes by analyzing the internal distribution of somatic missense mutations within functional regions of proteins [16]. MSEA, implemented through MSEA-clust and MSEA-domain, predicts cancer genes based on the presence of mutation hotspots in their functional domains or active sites [17]. iPAC utilizes protein tertiary structure to detect non-random somatic mutation clusters, enhancing the identification of oncogenic driver mutations [18]. OncodriveFML identifies drivers (genomic regions of interest) by comparing the observed average impact score on each region with the expected score resulting from sampling [19]. These methods have the advantage of identifying driver genes that undergo positive selection at the protein level rather than just the mutation level. The third category encompasses network-based methods, which aim to identify a set of interacting genes based on prior knowledge [21–26]. Network-based methods can identify driver genes that may not have a high mutation frequency but play regulatory roles in protein networks. However, a critical challenge for these methods lies in the completeness and accurate utilization of prior knowledge databases.

Similar to mutational heterogeneity, the functional impacts of mutations also exhibit heterogeneity (functional heterogeneity) due to various evolutionary conservation patterns [27, 28]. Specifically, mutations located in the same gene could have different functional impacts on the tumor. Several algorithms have been developed to evaluate functional impacts, e.g., MutationAssessor [28], SIFT [29], GERP [30], PolyPhen [31], and CADD [32]. Besides, many bioinformatics methods that are based on functional impact have also been deployed to prioritize candidate genes. However, these methods still face some limitations. Firstly, several of these methods cannot yield stable results regarding the number of identified drivers across different tumor types. For instance, e-Driver, MSEA, OncodriveFML, and iPAC recognize anywhere from no driver genes to hundreds of driver genes across 31 studied tumor types. Secondly, the lists of driver genes that these methods predict lack consistency [33, 34]. Moreover, there is a shortage of established models to evaluate the background functional impact for genes, which reflect the expected functional impact based on average values in a similar manner to background mutation frequency. Random sampling is a typical approach for obtaining a null hypothesis in function-based methods [16, 17, 19, 35].

We aimed to identify cancer-associated genes by introducing a generalized linear regression model (GLM) with shrinkage and double-weighted strategies for identifying cancer driver genes with functional impact (GSW-FI). Specifically, our model employs a GLM to predict the background functional impact score (BFIS) of each gene, utilizing twelve genomic features that are relevant to somatic mutations and protein functional impact as explanatory factors. Furthermore, we implemented a shrinkage strategy on estimated BFIS to smooth out estimations and reduce deviation issues. Our shrinkage strategy takes advantage of neighboring gene information to improve estimation stability. Additionally, we used a double-weighted strategy composed of two separate weight tactics to assign moderate levels of importance to genes. With these strategies, GSW-FI can provide clear evaluations of BFIS and rational observed functional impact scores (FIS) for genes. Finally, we conducted a comparison between the observed FIS and the distribution of BFIS to pinpoint genes exhibiting significant bias, thereby identifying them as potential cancer driver genes. Our comprehensive evaluation, utilizing unbiased benchmarks proposed by prior research [36, 37], consistently demonstrated the superior performance of GSW-FI compared to ten other driver gene prediction methods on 31 TCGA datasets.

## Methods

### The workflow of GSW-FI

**Fig. 1:**
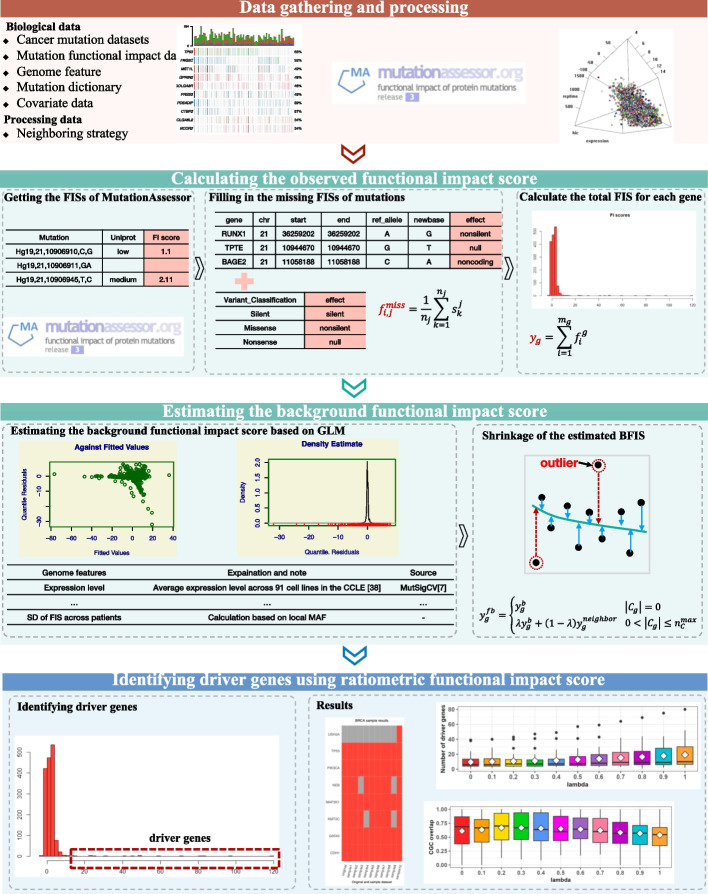
Workflow of GSW-FI

The proposed GSW-FI model is composed of four procedures: data gathering and preprocessing, calculating the observed functional impact score, estimating the background functional impact score, and identifying driver genes using ratiometric functional impact score, as shown in Fig. 1. The data preprocessing and analysis process primarily employed several R packages, namely plyr, stringr, MASS, and gamlss. Furthermore, the source code for GSW-FI can be freely accessed at https://github.com/bioinformatics-xu/GSW-FI.

### Data gathering and preprocessing

#### Mutation datasets from TCGA

We used the Mutation Annotation Format (MAF) files retrieved from TCGA (https://tcga-data.nci.nih.gov/tcga/) to conduct the driver gene analysis. For each mutation in the MAF file, we extracted information, including the patient with the mutation, chromosome start and end sites, the affected gene, reference and alternate nucleotide sites, and the type of variation. Besides, our method’s performance was evaluated using datasets from 31 TCGA projects, with detailed information available in Additional file 2: Table S1.

#### Functional impact data of mutations

To assess the functional impact of mutations, we utilized the Functional Impact Scores (FISs) obtained from MutationAssessor [28]. MutationAssessor evaluates the impact of mutations by considering the evolutionary conservation of the affected amino acid in protein homologs. The “MA scores rel3 hg19 full” file, which contains the mutation impacts for the hg19 reference genome (chromosomes 1 to 22, M, X, and Y), was used in this study and obtained from the MutationAssessor website (http://mutationassessor.org/r3/). In addition to MutationAssessor, other methods such as SIFT [29], PolyPhen [31], and CADD [32] can be used to calculate the FISs and are compatible with our research.

#### Genome feature

Genomic features, such as expression level, DNA replication time, and 3D chromatin interaction capture (HiC) features, have been shown to be relevant to mutation frequency [7]. We hypothesized that the FISs of genes are also related to certain genomic features, which may contribute to their ability to trigger cancer. To build our GLM model, we designed a comprehensive set of twelve predictive genomic features, described in Table 1.

**Table 1:** Description of 12 predictive genome features

Genome features	Explaination and note	Source
Expression level	Average expression level across 91 cell lines in the CCLE [38]	MutSigCV[7]
DNA replication time	Scale of 100 (early) to 1500 (late)	MutSigCV [7]
HiC-derived metric	The chromosomal compartment localization of the gene	MutSigCV [7]
Length of genomic regions	Combined coding regions	WITER [39]
Constraint score for non-synonymous mutations	Normalized student residues	Samocha et al. [40]
Expression hubs	Hubness in a gene expression network	MERGE [41]
Known regulators	Gene’s known regulatory role based on gene annotation databases	MERGE [41]
Genomic CNV	Genomic CNV status	MERGE [41]
Methylation	Methylation status	MERGE [41]
Total mutation number among patients	Calculation based on local MAF	–
Harmful mutation number among patients (null and nonsilent effects)	Calculation based on local MAF	–
Standard deviation of FISs across patients	Calculation based on local MAF	–

To validate the features with missing values, we employed a neighboring strategy [42, 43] for imputing the missing data as described below:

1. Let $v^*_{k,g}$ denote the missing value for gene *g* in feature *k*. The Euclidean distance between gene *i* and *j* in feature space (excluding feature *k*) is calculated as:
  1 $$\begin{aligned} D_{i,j} = \sqrt{\displaystyle \sum _{l \ne k} (v_{l,i} - v_{l,j})^2}, \end{aligned}$$
  where $v_{l,i} (v_{l,i})$ represents the value of feature *l* for gene *i*(*j*). Let $N_{g_k}$ denote the set of adjacent genes to gene *g* in feature *k*. The genes in $N_{g_k}$ must satisfy two criteria:
  2 $$\begin{aligned} \forall {(m \in N_{g_k}, n \not \in N_{g_k})}{(D_{g,m} \le D_{g,n})},{} & {} \end{aligned}$$
  3 $$\begin{aligned}{} & {} \left| N_{g_k}\right| = K, \end{aligned}$$
  where *K* is the size of $N_{g_k}$, which has been set to 100 in this study.
2. Validation of $v^*_{k,g}$ is performed using feature *k* of genes in the set $N_{g_k}$ as follows:
  4 $$\begin{aligned} v^*_{k,g}= \dfrac{1}{K} \sum _{t \in N_{g_k}}v_{k,t}. \end{aligned}$$
3. Each value of gene *g* in feature *k* is standardized by subtracting the mean and dividing by the standard deviation across genes.

### Calculating the observed functional impact score

In this research, we utilize MutationAssessor to assign the Functional Impact Scores (FISs) for mutations. The assignment of FISs consists of three steps:

1. Obtaining the FISs from MutationAssessor. Each mutation in the MAF file was matched to the mutations in “MA scores rel3 hg19 full” files using information such as chromosome, mutation site, reference base, and alteration base.
2. Filling in the missing FISs of mutations. The variation classification (such as silent, synonymous, nonsense, nonstop, in frame deletion) in the MAF file were mapped to the corresponding mutation effect (silent, nonsilent, noncoding, and null) based on the mutation type dictionary file [7].

The missing FISs of mutations can be filled by the average FIS of the corresponding mutation effect. Specifically, the FIS of mutation *i* with effect *j* is filled by

5 $$\begin{aligned} f_{i,j}^{miss} = \dfrac{1}{n_j}\displaystyle \sum _{k=1}^{n_j}s_k^j, \end{aligned}$$

where $n_j$ is the number of mutations with effect *j*, and $s_k^j$ represents the FIS of mutation *k* with effect *j*.

Due to the potential presence of missing values in MutationAssessor, it may not always be feasible to calculate the average FIS for mutations with effect *j*. Hence, imputing missing values with Eq. (5) is not universally applicable. In such cases, we propose the following approach to fill the FIS for each specific mutation *i* with effect *j*:

6 $$\begin{aligned} f_{i,j}^{miss} = {\left\{ \begin{array}{ll} 0 &{} \text{ mutation } \text{ effect } j \text{ is } \text{ silent, } \\ 1 &{} \text{ mutation } \text{ effect } j \text{ is } \text{ noncoding, } \\ 2 &{} \text{ mutation } \text{ effect } j \text{ is } \text{ nonsilent, } \\ 3 &{} \text{ mutation } \text{ effect } j \text{ is } \text{ null. } \end{array}\right. } \end{aligned}$$

(3) Calculating the total FIS for each gene. The total FIS for gene *g* is calculated by
  7 $$\begin{aligned} y_g = \displaystyle \sum _{i=1}^{m_g}f_i^g, \end{aligned}$$
  where $m_g$ is the number of mutations in gene *g*, and $f_i^g$ is the FIS of mutation *i* in gene *g*.

### Estimating the background functional impact score

#### Estimating the background functional impact score based on generalized linear regression model

*Generalized linear regression model* We have developed a GLM model to estimate the background functional impact of genes. The model uses FIS as the dependent variable and incorporates 12 genomic features as independent variables, as listed in Table 1. Let $\{y_g | g = 1, 2, \ldots , N \}$ denote the observed FIS values, where *N* is the total number of genes under study. Considering that FIS values are real continuous, a normal distribution generalized linear model to identify each gene is proposed

8 $$\begin{aligned} g(\mu _g) = \varvec{x}_g^T \varvec{\beta }, \end{aligned}$$

where $\varvec{x}_g = \{1, x_{g1}, x_{g2}, \ldots , x_{gp} \}^T$ is a $(p + 1) \times 1$ gene feature vector, and $\varvec{\beta } = \{\beta _{0}, \beta _{1}, \ldots , \beta _{p} \}^T$ is a $(p + 1) \times 1$ regression coefficient vector that captures the effects of gene features. $\mu _g$ represents a linear function of $p + 1$ features, and it is associated with $y_g$ through an identity link function *g*(.). Therefore, the GLM model is

9 $$\begin{aligned} \mu _g = \varvec{x}_g^T \varvec{\beta }. \end{aligned}$$

The distribution of $y_g$ depends on $\varvec{x}_g^T \varvec{\beta }$ and an unknown variance parameter $\epsilon _g$. The corresponding linear regression model is

10 $$\begin{aligned} y_g = \varvec{x}_g^T \varvec{\beta } + \epsilon _g, \end{aligned}$$

Here, $\{\epsilon _g|g=1, 2, \ldots , N\}$ are independent and identically distributed from a normal distribution with zero-mean and a standard deviation of $\sigma _0$, i.e., $\epsilon _g \sim \mathcal N(0,\sigma _0^2)$. Based on the above model assumptions,

11 $$\begin{aligned} y_g \sim \mathcal N(\varvec{x}_g^T \varvec{\beta },\sigma _0^2). \end{aligned}$$

*The background functional impact score* The regression coefficients $\varvec{\beta }$ and standard deviation $\sigma _0$ were estimated using the maximum likelihood method. For a detailed procedure, please refer to the Additional file 1. After obtaining the parameter $\varvec{\beta }$, the BFIS of gene *g* can be expressed by

12 $$\begin{aligned} y_g^{b} = \varvec{x}_g^T \varvec{\beta }. \end{aligned}$$

#### Shrinkage of the estimated background functional impact score

Shrinkage estimation, a useful method for correcting outliers, has been widely applied in genome research [44, 45]. Building on the assumption that FISs are associated with gene features, a shrinkage strategy was employed to refine the estimated BFIS.

*Building the functional impact score circle* In detail, the selection of neighbors in the FIS circle for gene *g* ($C_g$) should satisfy the following three criteria:

First, the closest neighboring genes in the feature space are chosen to be part of the circle:

13 $$\begin{aligned} \forall {(i \in C_g, j \not \in C_g)}{(D_{g,i} \le D_{g,j})}. \end{aligned}$$

Here, all gene features are utilized to define the circle and are scaled as described in the “*Genome feature*” section. The Euclidean distance between gene *i* and *j* is calculated using Eq. (1).

Second, all genes within the FIS circle should exhibit similarity to the gene under study in terms of functional impact scores. To determine this, the FISs of gene *g* ($y_g$) and its neighbors within the FIS circle ($y_i$) must pass a hypothesis test

14 $$\begin{aligned} {\begin{matrix} Q_{i,g}^{left} = &{} \mathcal N_C(y_g - y_i,0,1),\\ Q_{i,g} = &{} 2 \min \left( Q_{i,g}^{left}, 1-Q_{i,g}^{left} \right) ,\\ Q_{i,g} \le &{} 0.1. \end{matrix}} \end{aligned}$$

where $\mathcal N_C (x,0,1)$ represents the cumulative standard normal distribution.

Third, the number of neighbors in the FIS circle is limited by

15 $$\begin{aligned} \left| C_g\right| \le n_C^{max}, \end{aligned}$$

where $\left| C_g\right|$ denotes the number of neighbors in the FIS circle of gene *g*, and $n_C^{max}$ represents the maximum allowable number of neighbors. To strike a balance between computation complexity and obtaining sufficient information from neighbors, we have set $n_C^{max}=100$ for this research. It is worth noting that users can adjust the value of $n_C^{max}$ according to their specific dataset characteristics and requirements.

*Shrinking background functional impact scores through neighbor genes* Next, the BFIS of gene *g* was refined through a shrinkage strategy that incorporates the FISs of its neighboring genes. The influence of the neighbor genes on the BFIS is determined by their proximity to the gene under study in the feature space. The neighbor FIS for gene *g* is calculated by

16 $$\begin{aligned} y_g^{neighbor} = \dfrac{\displaystyle \sum _{k \in C_g}\left( \dfrac{y_k}{D_{k,g}}\right) }{\displaystyle \sum _{k \in C_g}\left( \dfrac{1}{D_{k,g}}\right) }. \end{aligned}$$

The resulting BFIS for gene *g* after applying shrinkage, is determined by:

17 $$\begin{aligned} y_{g}^{fb} = {\left\{ \begin{array}{ll} y_g^{b} &{} \left| C_g\right| = 0, \\ \lambda y_g^{b} + \left( 1-\lambda \right) y_g^{neighbor} &{} 0 < \left| C_g\right| \le n_C^{max}. \end{array}\right. } \end{aligned}$$

Here, $\lambda \in \left( 0,1\right)$ represents the weight coefficient that balance the impact of $y_g^b$ (original BFIS) and $y_g^{neighbor}$ (influenced by neighbor genes).

### Identifying driver genes using ratiometric functional impact score

#### Determining two weight coefficients of observed functional impact score

The proportion of harmful mutations to total mutations for a gene is a crucial metric for evaluating its destructiveness. The ratiometric method that assesses the composition of mutations in a gene to identify driver genes have been studied extensively [46–48]. We have further introduced the ratiometric method to calculate observed FIS and proposed the double-weighted strategy.

The double-weighted strategy involves two weights. The first weight is the proportion of harmful mutations to total mutations in a gene, indicating the degree of harmfulness of mutations. This weight is calculated by

18 $$\begin{aligned} w_1^g = \dfrac{m_g^{harm}}{m_g^{total}}, \end{aligned}$$

where $m_g^{harm}$ is the number of mutations with harmful effects in gene *g*; $m_g^{total}$ is the total number of mutations in gene *g*. For one gene, $m_g^{harm} \le m_g^{total}$, thus $w_1^g \in [0,1]$.

The second weight, denoted as $w_2^g$, is calculated as the exponential proportion of harmful mutations to the total number of samples, allowing for the effective integration of information regarding the number of harmful mutations. The calculation of this weight is

19 $$\begin{aligned} w_2^g = \exp \left( \dfrac{m_{g}^{harm}}{M} \right) , \end{aligned}$$

where *M* is the total number of samples, and $m_{g}^{harm}$ is the number of samples with harmful mutations in gene *g*. Normally, $0 \le m_{g}^{harm} \le M$, so $w_2^g \in [1,e]$. The weighted observed FIS of gene *g* is then given by

20 $$\begin{aligned} y_g^{w} = w_1^g w_2^g y_g. \end{aligned}$$

The first weight enhances the FIS for genes with a higher rate of harmful mutations, while the second weight amplifies the FIS for genes with a larger number of deleterious mutations.

#### Identifying driver genes

For genes that do not have any harmful mutations, a *p*-value of 1 is assigned. Conversely, for genes with harmful mutations, the weighted observed FIS is compared against the final BFIS. Essentially, the *p*-value for each gene represents the probability of obtaining a weighted observed FIS ($y_g^{w}$) equal to or greater than its value by chance, assuming the null distribution. The null distribution is assumed to be normal, with the final BFIS ($y_g^{fb}$) serves as the mean and the estimated variance $\sigma _0$ as the covariance. Finally, the Benjamini-Hochberg false discovery rate algorithm is utilized to calculate the *q*-value for each gene. Genes with a *q*-value $\le 0.05$ are identified as significant driver genes.

### Driver genes prediction methods and evaluation metrics

GSW-FI has been compared to ten other commonly used methods for identifying cancer-associated genes in 31 TCGA datasets. These methods include Dendrix[49], DriverNet[13], e-Driver[16], iPAC, MEMo[50], MSEA, MutSigCV, DriverML [37], OncodriveFML [19], and rDriver [20]. The driver gene lists of these methods were obtained from DriverDBv2 [33] and DriverML, and GSW-FI was run on the same datasets.

Evaluating the performance of these methods is challenging due to the absence of a universally accepted standard. However, several evaluation metrics have been employed to measure driver gene prediction performance, which serve as valuable indicators [36, 37, 39]. The high percentages of overlap with well-established databases indicate excellent performance in identifying driver genes [13, 51]. Therefore, one of the evaluation metrics used in this study is the overlap with three well-established databases, CGC [52], Mut-driver [53], and HiConf [54]. CGC is a widely recognized database that identifies genes implicated in oncogenesis, providing information on sequence alterations, cancer types, and protein domains associated with cancer genes. The CGC database currently includes 738 genes (as of October 7, 2023) and can be accessed at https://cancer.sanger.ac.uk/census#cl_search. Mut-Driver aims to identify driver mutations and genomic alterations in human cancer, encompassing 125 genes. HiConf is a panel of statistical tests that effectively detects oncogenes and tumor suppressor genes in cancer based on patient bias and truncation event rate, covering 99 genes (https://github.com/Bose-Lab/Improved-Detection-of-Cancer-Genes). The overlap fraction (OF) with these three databases is defined as the proportion of genes in the database to all the identified genes. It is calculated as:

21 $$\begin{aligned} \textrm{OF}_j = \frac{|O_j|}{|A_j|}. \end{aligned}$$

Here, $\textrm{OF}_j$ represents the overlap fraction of cancer type *j*. The set $O_j$ contains genes that are identified by the evaluated method in cancer type *j* and are also present in the database. The set $A_j$ includes all genes identified by the evaluated method in cancer type *j*. The notation $|\cdot |$ denotes the cardinality of a gene set.

Another metric used is the ability to identify genes recognized as potential drivers by multiple methods [55]. For each evaluated method, genes that are predicted by at least one, two, and three other methods are included. These sets are denoted as $D_j^t (t = 1, 2, 3)$ for cancer type *j*, and they represent genes predicted by at least *t* other methods. The method consensus of the evaluated method, which represents the proportion of identified genes predicted by at least *t* other methods, is denoted as $\textrm{MC}_j^t$. It is calculated as follows:

22 $$\begin{aligned} \textrm{MC}_j^t = \frac{|D_j^t|}{|A_j|} \end{aligned}$$

The set $A_j$ is defined as in equation (21).

In addition to precision, it is crucial for methods to yield robust and stable results across different tumor types. The expectation is for methods to identify a moderate number of genes across various tumor types, with minimal drastic changes in the number of identified drivers between different tumors. Therefore, the standard deviation of the identified driver gene count across various tumor types is another metric used to assess the robustness of the methods in this research.

## Results

### Uncertainty analysis of GSW-FI

The proposed GSW-FI model for identifying driver genes incorporates shrinkage and double-weighted strategies. The dual weights are calculated from mutation data using Eqs. (18) and (19), and they do not affect the model’s stability. Please refer to the Additional file 1 for an analysis of the impact of these weights on the model. Additionally, we will perform an uncertainty analysis of the model with respect to both the shrinkage parameter $\lambda$ and sample noise.

#### The impact of $\lambda$ on performance

**Fig. 2:**
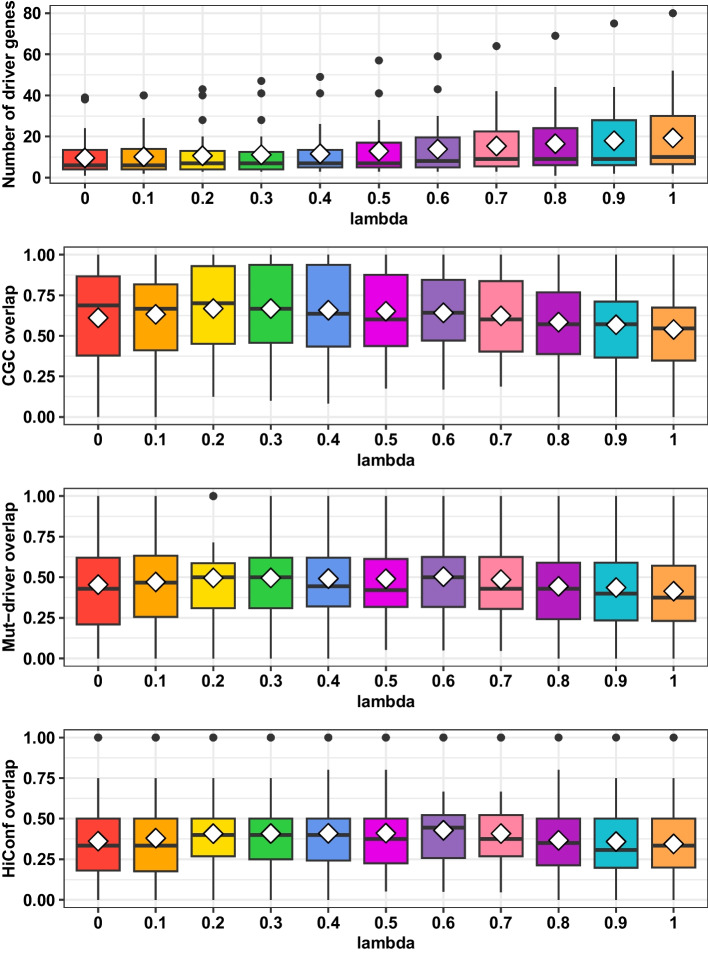
The performance of GSW-FI with $\lambda$ from 0 to 1 with a step size of 0.1. The white diamond represents the mean value

Parameter $\lambda$ directly determines the strength of shrinkage and affects the performance of GSW-FI. As $\lambda$ increases, the influence of a gene’ estimated FIS on the final BFIS becomes more significant while the impact from neighboring genes decreases. Choosing an appropriate $\lambda$ value helps control potential false positive predictions. We examined the sensitivity of GSW-FI across 31 datasets by incrementally varying $\lambda$ from 0 to 1 with a step size of 0.1. The analysis included the number of predicted driver genes and their overlap fractions (Eq. (21)) with three driver gene databases, i.e., CGC, Mut-Driver, and HiConf as shown in Fig. 2.

In general, larger $\lambda$ values lead to a higher number of predicted driver genes. For instance, in the CHOL dataset, the number of identified driver genes for different $\lambda$ values (ranging from 0 to 1) are as follows: (6, 6, 10, 11, 11, 16, 20, 25, 25, 28, 30). Moreover, when considering the overlap with driver gene databases, the advantage lies within the $\lambda$ range between 0.3 and 0.6. Across 31 datasets, the highest average overlap fractions for the three databases were obtained at $\lambda$ values of 0.3, 0.6, and 0.6, resulting in the corresponding average overlap fractions of 0.6675, 0.4281, and 0.5035, respectively. This analysis highlights the importance of selecting an appropriate $\lambda$ value that strikes a balance and integration between the estimated functional impact of a gene and its neighboring genes. Additionally, we conducted a comprehensive comparison utilizing an extensive range of $\lambda$ values (including 0, 0.5, and 1.0), compared to the outcomes obtained from other methods in the following sections.

#### The influence of sample noise on identifying driver genes

**Fig. 3:**
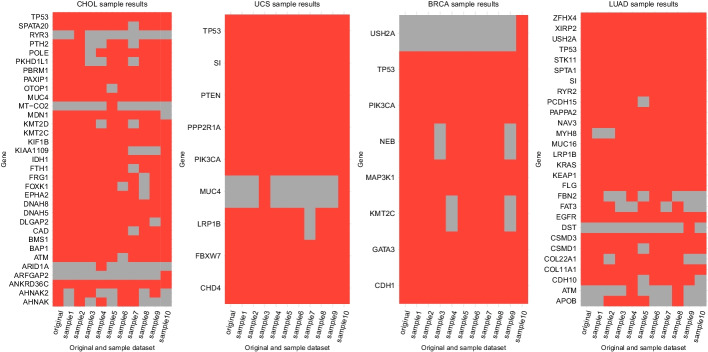
The overlap between the identified driver genes using original and subsampled datasets. The red color indicates that the genes on the x-axis were identified by GSW-FI when applied to the dataset on the y-axis, while the gray color represents that the genes on the x-axis were not identified using the dataset on the y-axis

To investigate the influence of sample noise on the identification of driver genes using GSW-FI, ten independent subsampling trials were conducted on four datasets (CHOL and UCS with small sample sizes, BRCA and LUAD with large sample sizes). In each trial, we use GSW-FI to identify driver genes and compare them with the results obtained using the original dataset. The overlap between the driver genes identified using the subsampled datasets and the driver genes identified using the original dataset are presented in Fig. 3. Specifically, we measure the overlap using the Jaccard similarity coefficient, which represents the ratio of the intersection of two sets to their union. The average Jaccard similarity coefficients for ten subsamples of these four datasets are 0.8706, 0.9653, 0.9304, and 0.8922, respectively. Our analysis indicates that GSW-FI is highly resilient to sample noise, as evidenced by the significant overlap between the driver genes identified from the subsampled datasets and the original dataset. Furthermore, the overlap tends to increase with larger dataset sizes, indicating that our proposed model may benefit from larger sample sizes.

### Biological pathway analysis for the identified driver genes by GSW-FI

We conducted the biological pathway analysis on the identified driver genes by GSW-FI ($\lambda = 0.5$) using DAVID [56]. The involved biological pathways of 31 driver gene set identified by GSW-FI have been summarized in Additional file 3: Table S2. With the exception of THYM (3 genes) and PRAD (5 genes), where a smaller number of genes were identified, GSW-FI exhibited a notable enrichment of driver genes within significant signaling pathways across the majority of datasets. Besides, a higher number of driver genes identified from the 31 datasets consistently led to more significant enrichment in important signaling pathways, as indicated by the improved pathway annotation results and significant FDR values. As an example, the UCEC dataset yielded the identification of 41 driver genes, which exhibited significant enrichment across 64 signaling pathways (FDR<0.05). Table 2 displays the annotation results of signaling pathways using LAML, BRCA, and LUAD datasets as examples. Specifically, in the LAML dataset, 11 genes including *CEBPA*, *DNMT3A*, *FLT3*, *IDH1*, *IDH2*, *NPM1*, *RUNX1*, *TET2*, *U2AF1*, *NRAS*, *TP53* were identified. These genes were found to be involved in critical pathways such as central carbon metabolism in cancer, acute myeloid leukemia, transcriptional misregulation in cancer, pathways in cancer, and chronic myeloid leukemia pathways, which are well-known to be associated with acute myeloid leukemia (LAML) cancer.

**Table 2:** Functional annotation results for identified driver genes by GSW-FI

Dataset	Terms	Genes	FDR
	hsa05230:Central carbon metabolism in cancer	*NRAS*, *FLT3*, *IDH1*, *IDH2*, *TP53*	3.1329E-05
	hsa05221:Acute myeloid leukemia	*CEBPA*, *NRAS*, *FLT3*, *RUNX1*	0.0014
LAML	hsa05202:Transcriptional misregulation in cancer	*CEBPA*, *FLT3*, *TP53*, *RUNX1*	0.0214
	hsa05200:Pathways in cancer	*CEBPA*, *NRAS*, *FLT3*, *TP53*, *RUNX1*	0.0234
	hsa05220:Chronic myeloid leukemia	*NRAS*, *TP53*, *RUNX1*	0.0460
	hsa05213:Endometrial cancer	*PIK3CA*, *CDH1*, *TP53*	0.0271
BRCA	hsa05218:Melanoma	*PIK3CA*, *CDH1*, *TP53*	0.0271
	hsa04722:Neurotrophin signaling pathway	*MAP3K1*, *PIK3CA*, *TP53*	0.0493
	hsa05225:Hepatocellular carcinoma	*KEAP1*, *KRAS*, *TP53*, *EGFR*	0.0385
LUAD	hsa05223:Non-small cell lung cancer	*KRAS*, *TP53*, *EGFR*	0.0385
	hsa04151:PI3K-Akt signaling pathway	*STK11*, *KRAS*, *TP53*, *EGFR*	0.0435

### Harmful mutation ratio analysis for the identified driver genes by GSW-FI

As discussed in the section “Calculating the observed functional impact score”, mutations can be categorized into four effects: silent, non-silent, non-coding, and null. Among these, silent mutations (synonymous mutations) in the gene coding sequence and non-coding mutations in the flanking untranslated regions (UTRs) and intronic sequences are considered background mutations with a weak selective growth advantage for tumors [7]. In contrast, non-silent and null mutations that affect the amino acids of a protein or even cause frameshifts in the sequence have a significant impact on tumorigenesis. Previous studies have proposed various methods to quantify the selection in cancer genomes based on the ratio of non-synonymous to synonymous mutations. For example, Martincorena et al. [57] introduced the *dN*/*dS* index to evaluate selection in cancer genomes, where high *dN*/*dS* ratios indicate positive selection in tumor cells. Similarly, Lawrence et al. [7] and Tokheim et al. [36] used ratiometric features, such as the ratio of protein-affecting mutations to other mutations, to identify driver genes.

Motivated by these studies, we developed a ratiometric feature based on the ratio of harmful mutations (non-silent and null mutations) to total mutations in each gene. We calculated the ratios of harmful mutations for driver genes identified by GSW-FI model ($\lambda = 0.5$), as summarized in Additional file 4: Table S3. We found that the average harmful mutation ratio for driver genes was 0.8971 across 31 datasets, indicating that non-silent and null mutations play a crucial role in tumorigenesis. Notably, among the identified 399 driver genes across 31 datasets, 114 genes had a harmful mutation ratio of 1, indicating that all mutations in these genes were either non-silent or null mutations.

### Number of identified driver genes by 11 methods

The average number of predicted driver genes across 31 datasets by 11 methods ranged from 1 (MEMo) to 2918 (iPAC). The analysis above reveals that GSW-FI demonstrates an increasing number of predicted driver genes as $\lambda$ values grow larger. Here, we have chosen to perform the analysis using an intermediate value of $\lambda =0.5$. With this setting, GSW-FI identified a total of 399 driver genes across the analyzed datasets (ranging from 3 to 57), with some degree of overlap observed among different datasets. These 399 driver genes collectively involve 198 unique genes, indicating that certain genes are identified as drivers in multiple datasets. Specifically, GSW-FI detected fewer than ten genes in 13 datasets and identified more than 30 genes in two datasets. Other methods, such as Dendrix, e-Driver, rDriver, DriverNet, OncoDriveFML, and MutSigCV identified an average number of driver genes ranging from 10 to 50. To evaluate the range of the driver gene numbers, the standard deviation across the 31 datasets was calculated. A significant standard deviation suggests instability in the method’s results, which may potential concerns about the underlying algorithm. It is worth noting that MEMo, rDriver, and GSW-FI emerged as the top three methods with standard deviations of less than 15, indicating their relative robustness across the 31 datasets.

### Overlap analysis of predicted driver genes with CGC, Mut-Driver, and HiConf

**Fig. 4:**
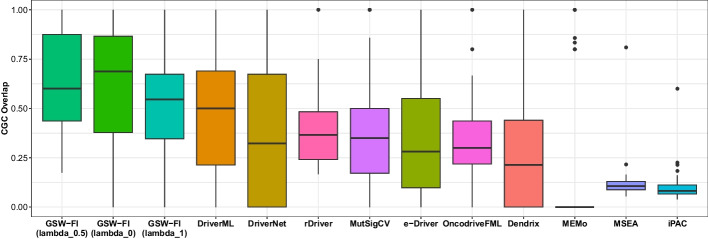
The overlap fractions with CGC databases of 11 methods across 31 TCGA datasets ($\lambda = 0, 0.5, 1$)

**Fig. 5:**
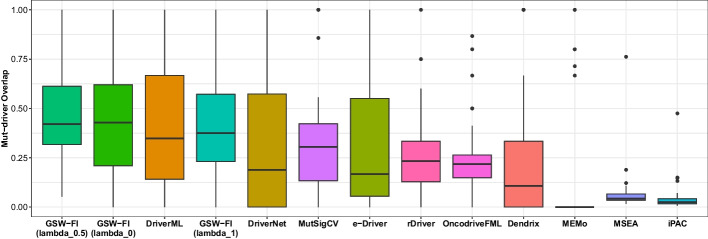
The overlap fractions with Mut-Driver databases of 11 methods across 31 TCGA datasets ($\lambda = 0, 0.5, 1$)

**Fig. 6:**
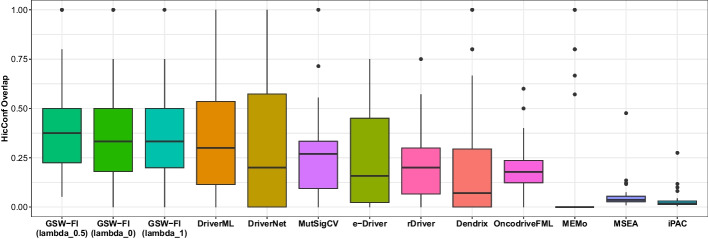
The overlap fractions with HiCinf database of 11 methods across 31 TCGA datasets ($\lambda = 0, 0.5, 1$)

The fractions of overlap between the identified genes and the CGC, Mut-driver, and HiConf databases for the 11 evaluated methods across 31 TCGA datasets are depicted in Figs. 4, 5, 6, as well as detailed in Additional files 5, 6, 7. Reffering to [37], any method that predicted less than three genes in a dataset was assigned a value of zero. The methods in the figures are sorted from left to right based on their overall mean across the 31 datasets.

When compared to the CGC database (Fig. 4), GSW-FI showed the highest overlap, with average fractions of 61.06%, 65.13%, and 53.86% for lambda values of 0, 0.5, and 1, respectively. The next three methods, DriverML, DriverNet, and rDriver, had average overlap fractions of 48.34%, 39.41%, and 38.18%, respectively. CoMDP and SCS, as their main focus is on identifying gene modules with high coverage and mutual exclusivity, exhibited less than 10% driver gene predictions on average in the CGC gene list. Regarding the Mut-driver (Fig. 5) and HiConf (Fig. 6) databases, which contain fewer genes than CGC, the overall average fractions are comparatively lower. GSW-FI also demonstrated the highest overlap in these databases, with average fractions of 45.44%, 49.01%, and 41.41% for Mut-driver, and 36.14%, 41.10%, and 34.50% for HiConf, corresponding to lambda values of 0, 0.5, and 1, respectively. In the Mut-driver database, the top three methods were DriverML, DriverNet, and MutSigCV, with percentages of 41.48%, 33.51%, and 31.73%, respectively. In the HiConf database, the top three methods were DriverML, DriverNet, and MutSigCV, with percentages of 34.00%, 32.37%, and 26.48%, respectively. iPAC and MSEA predicted less than 10% of driver genes on average in the Mut-driver and HiConf databases. Overall, GSW-FI achieved the highest average percentage of predicted driver genes among the CGC, Mut-driver, and HiConf databases.

### Assessing the consensus among different methods in driver gene prediction

**Fig. 7:**
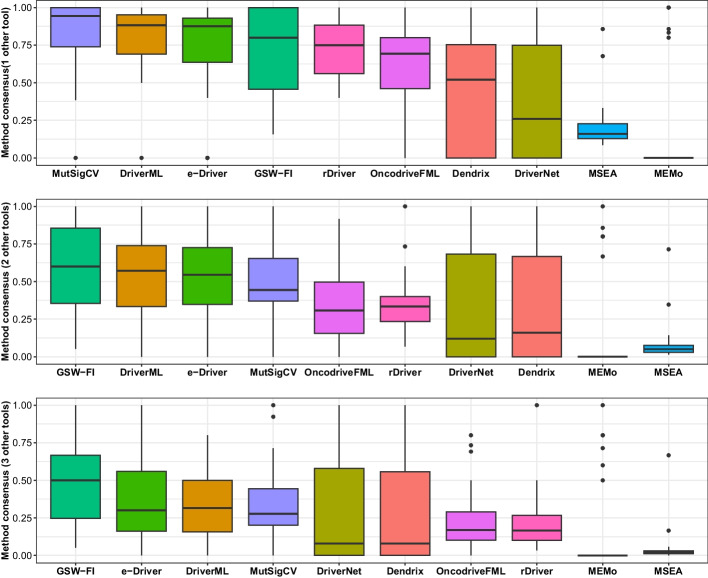
Method consensus results of methods on 31 TCGA datasets ($\lambda = 0.5$)

Referring to the works of [36, 58], we evaluated the level of consensus among different methods by examining the fraction of driver genes that were also predicted by one, two, and three additional methods (using GSW-FI with $\lambda$=0.5). The method consensus results, calculated using Eq. (22), are presented in Fig. 7. iPAC was excluded from the evaluation due to potential bias caused by the large number of predicted driver genes. Overall, GSW-FI, DriverML, MutSigCV, and e-Driver demonstrated the highest consistency among the methods. When considering driver genes identified by at least one other method, MutSigCV, DriverML, and e-Driver achieved average method consensus rates of 83.82%, 80.02%, and 73.54%, respectively, across 31 datasets. Additionally, all genes identified by GSW-FI in 12 datasets, MutSigCV in 10 datasets, and DriverML in 7 datasets were predicted by at least one other method. On average, 58.78% and 49.33% of genes identified by GSW-FI across the 31 datasets were also identified by at least two or three other methods, respectively. Furthermore, the method consensus rates noticeably decreased when considering genes identified by at least two and three other methods. For DriverML, e-Driver, and MutSigCV, the average fractions of genes across the 31 datasets identified by at least two and three other methods were (52.93%, 51.74%, 51.57%) and (34.85%, 36.84%, 33.24%), respectively.

### Overall performance

The efficiencies of 11 methods were evaluated by assessing their overlap fraction with three widely-confirmed driver databases, agreement with a consensus gene list of driver genes predicted by at least two other methods (including iPAC), and the standard deviation of the identified driver gene number across cancer types. To better understand the overall performance of these methods, we have summarized the results across 31 datasets in Table 3. Considering the overlap fraction with driver databases and method consensus, GSW-FI ranked first. The next three optimal methods were found to be DriverML, MutSigCV, and DriverNet. Additionally, robustness and stability are crucial characteristics of these methods. MEMo, rDriver, and GSW-FI demonstrated high robustness with minimal variation in the number of identified driver genes. However, MEMo and rDriver showed lower performance in terms of overlap with known databases and method consensus. In summary, GSW-FI outperformed the other methods by providing a comprehensive evaluation of accuracy and robustness.

**Table 3:** Overall performance of 11 methods

Methods	sd of driver gene number	CGC overlap (%)	Mut-driver overlap (%)	HiConf overlap (%)	Method consensus (%)	CGC rank	Mut-driver rank	HiConf rank	Method consensus rank	Average rank
Dendrix	38.78	29.22	25.04	21.49	39.83	8	8	7	7	7.50
MutSigCV	106.26	37.05	31.73	26.48	66.72	5	4	4	3	4.00
MEMo	3.02	17.07	16.07	15.61	17.71	9	15	9	10	10.75
DriverNet	44.02	39.41	33.51	32.37	36.76	3	3	3	8	4.25
e-Driver	42.73	36.07	29.57	25.00	61.80	6	5	5	4	5.00
iPAC	3662.54	11.35	5.31	3.55	7.2	11	11	11	11	11.00
MSEA	355.44	13.35	7.83	5.82	14.88	10	10	10	9	9.75
DriverML	251.56	48.34	41.48	34.00	73.99	2	2	2	1	1.75
OncodriveFML	69.71	33.93	26.18	19.17	53.55	7	7	8	5	6.75
rDriver	9.00	38.18	27.55	21.80	46.40	4	6	6	6	5.50
GSW-FI ($\lambda =0$)	9.36	63.26	45.44	36.14	–	–	–	–	–	–
GSW-FI ($\lambda =0.5$)	12.24	65.13	49.01	41.10	67.07	1	1	1	2	1.25
GSW-FI ($\lambda =1$)	18.25	53.86	41.41	34.50	–	–	–	–	–	–

$\bullet$ “sd of driver gene number” is the standard deviation of the identified driver gene number across cancer types

## Discussion

We have developed GSW-FI, a computational method for identifying cancer-associated genes that exhibit substantial functional impacts. GSW-FI was validated on 31 TCGA datasets, demonstrating robustness against data noise and accurate identification of driver genes. It exhibited a high level of overlap with established driver gene databases and showed excellent consistency with other methods. Furthermore, the biological pathway analysis has provided insights into potential biomarkers and therapeutic targets. For instance, in the case of lung adenocarcinoma (LUAD), the identified genes *KEAP1*, *KRAS*, *TP53*, *EGFR*, and *STK11* were found to be enriched in Hepatocellular carcinoma, Non-small cell lung cancer, and PI3K-Akt signaling pathway. These genes play crucial roles in the development of lung adenocarcinoma and have been validated as important biomarkers and therapeutic targets [59, 60].

Two benchmarks for evaluating new methods include the capability to accurately reproduce a significant number of extensively studied cancer genes documented in databases (such as CGC), as well as the ability to identify the core gene set predicted as driver genes by established methods. The methods that received the strongest support based on the criteria were GSW-FI, along with three other well-established methods: DriverML, MutSigCV, and DriverNet. The data presented in Table 3 clearly demonstrates that our proposed GSW-FI outperforms other methods in terms of overlap with respect to the driver gene databases. It secures the top position in overlap for all three databases: CGC, Mut-driver, and HiConf. Moreover, the gaps between GSW-FI and the second-ranked method are significant, with margins of 16.79%, 7.53%, and 7.1% respectively. Additionally, DriverML, GSW-FI, and MutSigCV are the top three methods that exhibit significantly greater overlap with other methods, with method consensus ranging from 66.72% to 73.99%. The advantage of GSW-FI, DriverML lies in their incorporation of the functional impact of gene mutations (i.e. functional heterogeneity), which enables a more comprehensive assessment of their significance as driver genes. On the other hand, DriverNet identifies likely driver mutations by analyzing their impact on mRNA expression networks. As for the renowned research method MutSigCV, it establishes the background mutation rate for each gene based on mutational heterogeneity.

In our framework, we faced some limitations. Firstly, we encountered missing values in MutationAssessor, which were essential for calculating functional impact scores. To ensure a more reliable and intelligent investigation of the functional impact of each gene in future research, it is critical to develop an informed approach. Additionally, we hypothesize that there might be a hidden correlation between the functional impacts of adjacent mutations that occur in neighboring chromosomal sites [61]. To effectively model the FISs of genomic regions of interest, we plan to employ methods such as the Hidden Markov Model in our forthcoming research efforts. These methods can deduce a series of states based on observed data. Addressing these issues will contribute to a more comprehensive understanding of the functional impacts of genes in cancer progression.

## Conclusions

In conclusion, our computational method GSW-FI has demonstrated its effectiveness in identifying cancer-associated genes with significant functional impacts. By incorporating gene features associated with functional impact scores (FIS) and utilizing advanced strategies such as double-weighted and shrinkage strategies, GSW-FI improves the precision and reliability of assessing gene functional impacts in relation to cancer. The validation of GSW-FI on TCGA datasets has shown its robustness against data noise and its accurate identification of driver genes. It exhibits a high level of overlap with established driver gene databases and demonstrates excellent consistency with other methods. The biological pathway analysis has provided valuable insights into potential biomarkers and therapeutic targets for specific cancer types, such as lung adenocarcinoma.

### Supplementary Information

**Additional file 1**. Supplementary Material for GSW-FI.

**Additional file 2**. **Table S1**: Summary of 31 TCGA datasets.

**Additional file 3**. **Table S2**: The involved biological pathways of 31 driver gene set identified by GSW-FI.

**Additional file 4**. **Table S3**: The ratios of harmful mutations for driver genes identified by GSW-FI in 31 datasets.

**Additional file 5**. **Table S4**: The fractions of overlap between the identified genes and the CGC databases for the 11 evaluated methods across 31 TCGA datasets.

**Additional file 6**. **Table S5**: The fractions of overlap between the identified genes and the MutDriver databases for the 11 evaluated methods across 31 TCGA datasets.

**Additional file 7**. **Table S6**: The fractions of overlap between the identified genes and the HiConf databases for the 11 evaluated methods across 31 TCGA datasets.

## Data Availability

The datasets generated and analysed during the current study are available in the TCGA repository, https://tcga-data.nci.nih.gov/tcga/.

## Abbreviations

NGS: Next-generation sequencing
ICGC: International Cancer Genome Consortium
TCGA: The cancer genome altas
GLM: Generalized linear regression model
FIS: Functional impact score
BFIS: Background functional impact scores
MAF: Mutation annotation format
CNA: Copy number variation
