# Supplementary material for: Gsw-fi: a GLM model incorporating shrinkage and double-weighted strategies for identifying cancer driver genes with functional impact
Source: BMC Bioinformatics. 2024 Mar 6;25:99. doi: 10.1186/s12859-024-05707-8 (PMC10916024; doi:10.1186/s12859-024-05707-8)
Supplement: Supplementary file 1 — Additional file 1. Supplementary Material for GSW-FI. [file 12859_2024_5707_MOESM1_ESM.pdf]

Supplementary of GSW-FI

1. The maximum likelihood estimation for regression

We have developed a GLM model to estimate the background functional impact of genes. Let $\{y_g|g = 1, 2, \cdots, N\}$ denote the observed FIS values, where N is the total number of genes under study. The distribution of y_g depends on $\boldsymbol{x}_g^T\boldsymbol{\beta}$ and an unknown variance parameter ϵ_g. The corresponding linear regression model is

$$y_g = \boldsymbol{x}_g^T\boldsymbol{\beta} + \epsilon_g, \tag{1}$$

Here, $\{\epsilon_g|g = 1, 2, \cdots, N\}$ are independent and identically distributed from a normal distribution with zero-mean and a standard deviation of σ_0, i.e., $\epsilon_g \sim \mathcal{N}(0, \sigma_0^2)$. Based on the above model assumptions,

$$y_g \sim \mathcal{N}(\boldsymbol{x}_g^T\boldsymbol{\beta}, \sigma_0^2). \tag{2}$$

The feature matrix is denoted as $X = (\boldsymbol{x}_1, \boldsymbol{x}_2, \cdots, \boldsymbol{x}_N)^T$. The corresponding response vector is denoted as $\boldsymbol{y} = (y_1, y_2, \cdots, y_N)^T$. The likelihood function can be expressed as follows:

$$\begin{aligned} \mathcal{L}(\boldsymbol{\beta}, \sigma_0|X, \boldsymbol{y}) &= \prod_{g=1}^{N} \frac{1}{\sqrt{2\pi}\sigma_0}\exp\left(-\frac{(y_g - \boldsymbol{x}_g^T\boldsymbol{\beta})^2}{2\sigma_0^2}\right), \\ &= \left(\frac{1}{\sqrt{2\pi}\sigma_0}\right)^N \exp\left(-\frac{1}{2\sigma_0^2}\sum_{g=1}^{N}\left(y_g - \boldsymbol{x}_g^T\boldsymbol{\beta}\right)^2\right). \end{aligned} \tag{3}$$

The corresponding log-likelihood function is

$$\log\mathcal{L}(\boldsymbol{\beta}, \sigma_0|X, \boldsymbol{y}) = -N\log\sqrt{2\pi} - N\log\sigma_0 - \frac{1}{2\sigma_0^2}\sum_{g=1}^{N}\left(y_g - \boldsymbol{x}_g^T\boldsymbol{\beta}\right)^2. \tag{4}$$

Consequently, the optimization model can be summarized as follows:

$$\max \ \log\mathcal{L} = -N\log\sqrt{2\pi} - N\log\sigma_0 - \frac{1}{2\sigma_0^2}\sum_{g=1}^{N}\left(y_g - \boldsymbol{x}_g^T\boldsymbol{\beta}\right)^2. \tag{5}$$

This is a convex optimization problem, and the optimal $\boldsymbol{\beta}$ can be obtained by

$$\frac{\partial\mathcal{L}(\boldsymbol{\beta}, \sigma_0|X, \boldsymbol{y})}{\partial\boldsymbol{\beta}} = -\frac{1}{\sigma_0^2}\sum_{g=1}^{N}\left(y_g - \boldsymbol{x}_g^T\boldsymbol{\beta}\right)\boldsymbol{x}_g = 0. \tag{6}$$

The optimal estimation for $\boldsymbol{\beta}$ is as follows:

$$\widehat{\boldsymbol{\beta}} = \left(X^TX\right)^{-1}X^T\boldsymbol{y} \tag{7}$$

σ_0 is calculated by

$$\widehat{\sigma}_0 = \sqrt{\frac{1}{N}\sum_{g=1}^{N}\left(y_i - \boldsymbol{x}_g^T\boldsymbol{\beta}\right)^2}. \tag{8}$$

2. The analysis of the impact of double-weighted on GSW-FI model

The proposed GSW-FI model utilizes an effective double-weighted strategy to calculate the Functional Impact Score (FIS) for genes. The first weight, denoted as w_1^g, represents the proportion of harmful mutations to total mutations in the range of [0,1], indicating the degree of harmflessness of mutations. The second weight, denoted as w_2^g, is the exponential proportion of the number of mutations with harmful effects to the total number of samples. The w_1^g enhances the FIS for genes with a higher rate of harmful mutations, while the weight w_2^g amplifies the FIS for genes with a larger number of deleterious mutations.

Take the analysis of Adrenocortical carcinoma (ACC) as a example, we provide some key indicators for several genes in the following Table. When considering only w_1^g, the p-value for gene ZFPM1 is 0.1408. However, when both w_1^g and w_2^g are incorporated, the p-value becomes 0. By incorporating w_2^g, we are able to integrate information about the number of harmful mutations for genes, such as the 112 harmful mutations in ZFPM1. A similar situation also occurs in the gene ZNF517. Both of these genes have been confirmed to play a crucial role in ACC and other types of cancer[1-3]. On the other hand, if the ratio of harmful mutations is low, the p-value of gene will be adjusted into the non-significant direction, such as *KRTAP4-11* and *CCDC168*.

Table S 7: Explanation of double-weighted strategy

Genes	Number of Samples	Mutation Number	Harmful Mutation Number	w_1^g	w_2^g	p_{before}	p_{after}
ERCC2	90	19	19	1	1.235	8.3630e-06	3.4305e-14
MUC2	90	44	33	0.75	1.443	1.0182e-09	7.0821e-13
ZFPM1	90	112	112	1	3.471	0.1408	0
ZNF517	90	34	34	1	1.459	0.0390	1.1912e-07
KRTAP4-8	90	7	7	1	1.081	0.0162	0.0026
KRTAP4-11	90	22	17	0.773	1.208	0.4889	0.5908
CCDC168	90	10	4	0.4	1.045	0.1431	0.4669

p_{before} is the p-values before the application of the weighting, and p_{after} is the p-values after the application of the weighting.

[1] Rahane C S, Kutzner A, Heese K. Establishing a human adrenocortical carcinoma (ACC)-specific gene mutation signature[J]. Cancer Genetics, 2019, 230: 1-12.
[2] Liu X, Huang X, Bai Y, et al. Next-generation sequencing revealed recurrent ZFPM1 mutations in encapsulated papillary carcinoma of the breast[J]. NPJ Precision Oncology, 2021, 5(1): 42.
[3] Klebanov N, Artomov M, Goggins W B, et al. Burden of unique and low prevalence somatic mutations correlates with cancer survival[J]. Scientific reports, 2019, 9(1): 4848.
